# Important Medicinal Plant Families in Thailand

**DOI:** 10.3389/fphar.2019.01125

**Published:** 2019-09-25

**Authors:** Methee Phumthum, Henrik Balslev, Anders S. Barfod

**Affiliations:** Ecoinformatics and Biodiversity, Department of Bioscience, Aarhus University, Aarhus, Denmark

**Keywords:** ethnomedicine, family importance, Bayesian analysis, binomial analysis, linear regression, relative regression residual

## Abstract

Throughout the world, surveys have been conducted at the country level to answer research questions pertaining to ethnomedicinal usage patterns. This study is focused on Thailand, which has never been surveyed systematically in this way. We mined 16,000 records of medicinal plant use from 64 scientific reports, which were published from 1990 to 2014. In total, 2,187 plant species were cited as being useful for medicinal purposes. The overall aim was to reveal the relative importance of the plant families for pharmacological research. To determine the most important medicinal plant families, we use a combination of three statistical approaches: linear regression, Binomial analysis, and Bayesian analysis. At the regional level, 19 plant families repeatedly stood out as being the most important from an ethnomedicinal perspective.

## Introduction

It is well-documented in the scientific literature that plants have been used for medicinal purposes for the past 60,000 years (Solecki, 1975). Still today, millions of people around the world depend on medicinal plants for their well-being (WHO, 2002). In the tropics, medicinal plants are often used on a regular basis in rural communities where pharmaceuticals are hard to obtain or even unavailable. This is in contrast to westernized societies where medicinal plants are typically used as an alternative or supplement to prescribed medicine (WHO, 2002). Medicinal plants are important for people, not only as a primary source of medicines but also as phytochemical building blocks for development of new drugs (Fabricant and Farnsworth, 2001). It is estimated that 67% of drugs used in chemotherapy are derived from natural products (Wangkheirakpam, 2018). This applies to the discoveries of active compounds such as vincristine (Raviña, 2011), taxol (Fischer et al., 2010), and artemisinin (Tu, 2011). Moreover, medicinal plants also offer an opportunity for rural dwellers to generate a cash income (EL-Hilaly et al., 2003).

There are several factors that influence how people select plants for medicinal purposes: tradition, efficacy, abundance, accessibility, doctrine of signatures, as well as taxonomic affiliation (Bennett and Husby, 2008). Interestingly, some plant families comprise higher proportion of medicinally useful species than expected from the null model of a linear relationship between species diversity and number of medicinally useful species. Traditional use of plants for medicines is not random but determined, to a certain degree, by taxonomic affiliation (Moerman, 1996; Bennett and Husby, 2008). Various statistical tools have been applied to test the relationship between species richness and number of medicinally useful species. In an ethnomedicinal study conducted in North America at the family level, Moerman (1996) plotted the relationship between the number of medicinally useful plant species and the number of native species. Although the classic regression model is a simple and common way to test patterns of medicinal knowledge (Moerman, 1996; Leonti et al., 2003; Bourbonnas-Spear et al, 2005; Douwes et al, 2008; Saslis-Lagoudakis et al., 2011), it suffers from a bias toward large families (Weckerle et al., 2011). Bennett and Husby (2008) used binomial analysis to overcome this bias in their test of the medicinal importance of Ecuadorian plant families. Bayesian analysis was recently introduced as an alternative to reveal over- and under-represented medicinal families (Weckerle et al., 2011). The technique performed particularly well for small data sets and showed similar results to those of the binomial analysis. Here, we combine all three above-mentioned statistical techniques and only refer to a plant family as important if all three statistical methods confirm that it includes a higher number of medicinal species than expected under the null model.

Studies around the world reveal that the medicinal importance of plant families is only partly overlapping across space. In North America, Moerman (1996) showed that *Asteraceae*, *Apiaceae*, *Ericaceae*, *Rosaceae*, and *Ranunculaceae* included higher numbers of medicinal species than expected based on their species richness. Likewise, in Ecuador medicinal plant species were overrepresented in *Zingiberaceae*, *Piperaceae*, *Lamiaceae*, *Amaranthaceae*, *Apiaceae*, and *Costaceae* (Bennett and Husby, 2008). In Campania, Italy, a study revealed that medicinal species were over-represented in *Lamiaceae*, *Rosaceae*, and *Malvaceae*. In contrast, the families *Orchidaceae*, *Caryophyllaceae*, *Poaceae*, and *Leguminosae* included fewer medicinally useful species than suggested by their total species numbers (Weckerle et al., 2011). A recent study from Hawaii revealed that not only *Leguminosae*, *Ericaceae*, *Malvaceae*, *Zingiberaceae*, and *Apocynaceae* but also *Poaceae* and *Cyperaceae* were the most important medicinal families (Ford and Gaoue, 2017). Similar studies were conducted in Belize (Bourbonnas-Spear et al., 2005), Mexico (Leonti et al., 2003), New Zealand (Saslis-Lagoudakis et al., 2011), Nepal (Saslis-Lagoudakis et al., 2011), and South Africa (Saslis-Lagoudakis et al., 2011). Recently, *Leguminosae*, *Lamiaceae*, *Euphorbiaceae*, *Apocynaceae*, *Malvaceae*, *Apiaceae*, and *Ranunculaceae* were listed as being the medicinally most important plant families on a worldwide scale (Kew, 2017).

In this study, we analyze and compare the medicinal usefulness of plant families across Thailand. The country boasts a high diversity of both plants and ethnic groups (Pooma and Suddee, 2014 and Phumthum et al., 2018). Thailand has traditionally been divided in seven phytogeographic regions in accordance with Tem Smitinand’s classification (Smitinand, 1958). Based on a meta-analysis of plant species distribution records, van Welzen et al. (2011) concluded that the country should be divided into four phytogeographic regions defined as areas with a “typical, unique, and distinct plant composition”: the southern, northern, eastern, and central regions. The overall plant diversity increases toward the Malay Peninsula, which has a less seasonal climate. The biodiversity in Thailand is generally under pressure from human activities, especially farming and urban development.

Within Thailand, more than 80 dialects are spoken, which belong to five linguistic families: Austronesian, Hmong-Mien, Sino-Tibetan, Tai-Kadai, and Austro-Asiatic (Premsrirat, 2004). The majority of the population in Thailand is referred to as local Thai. However, the population also comprises a number of ethnic minorities many of which have their main distribution outside Thailand in countries such as Myanmar (Moken, Kachin, and Taiyai), Yunnan (Mien, Haw, and Lue), and Tibet (Karen, Musue, and Lahu) (Phumthum and Balslev, 2018). It should be noticed that the comprehensive ethnomedicinal data underlying this study are biased toward the ethnic minorities, which have been visited most frequently by ethnobotanical researchers. Although the data may not reflect the average situation at the village level, it does capture all common medicinal plant uses (Phumthum et al., 2018) and provides insights into the diversity of plant used for medicinal purposes, not only in Thailand but also in the neighboring countries.

We will address the following research questions: which are the most important medicinal plant families locally and across Thailand? How do these compare with medicinally important families found elsewhere in the world?

## Materials and Methods

### Plant Use Records

Plant use records were extracted from 64 studies reported in the scientific literature (1 book, 2 reports, 29 journal articles, and 32 master and PhD theses; for more details on how we avoided data replication, criteria for including or excluding references, and a list of references, we refer to our previous publications (Phumthum and Balslev, 2018; Phumthum et al., 2018). Data sources such as pocketbooks, local knowledge, plant labels in parks and gardens, and news articles were avoided since they are often based on inaccurate vernacular names, and they are difficult to reproduce due to lack of metadata. We only included records that cited scientific names and plants that had been identified to the species. To be considered, the data should comply with recognized ethnobotanical collection standards. Most of the scientific reports underlying this study cited voucher specimens deposited in herbaria across Thailand. In cases where journal articles had been published based on Master and PhD studies i.e., Srithi et al. (2012), Srithi et al. (2012a), and Srithi et al. (2012b), we extracted the data strictly from the original thesis. We used data from studies conducted from 1990, when systematic ethnobotanical exploration began in Thailand, until 2014. All Masters and PhD theses were accessed *via* the website of the Thai Library Integrated System (https://www.thailis.or.th/tdc), which includes all Thai institutes of higher education. To gain insight into the variation of the most important medicinal plant families (MIMFs) at the subregional level, we divided the dataset in seven subsets based on geographic location of the villages in accordance with Phumthum and Balslev (2018).

### Plant Diversity

The *Thai Plant Names* book provides list of all plant species in Thailand (Pooma and Suddee, 2014). We updated the taxonomy using *The Plant List* website (www.theplantlist.org) and follow the family names used on the *Angiosperm Phylogeny* website (www.mobot.org). We identified no discrepancies except for the families *Lamiaceae* and *Leguminosae* on *The Plant List* website, which were named *Labiatae* and *Fabaceae* on *Angiosperm Phylogeny* website. We followed the former. The complete version of the list after updating included 9,793 plant species representing totally 276 families in Thailand. To avoid bias and random noise, we removed from the dataset all families with less than 10 species in Thailand. This reduced the number of families included in our analysis to 115 (**Table 1**) and the number of species to 9,097.

**Table 1:** Medicinal importance of all plant families in Thailand containing more than 10 species as revealed by three statistical approaches: regression residual analysis, binomial analysis and Bayesian analysis.

Family	T	M	Method					
			Regression		Binomial		Bayesian	
			P1	R	P2	Prob.	inf.	sup.
*Acanthaceae*	169	52	30	0.79*	38	9.53E-03*	0.2430*	0.3811
*Achariaceae*	11	2	9	−0.76	2	7.51E-01	0.0549	0.4841
*Amaranthaceae*	33	19	12	0.67*	7	1.62E-05*	0.4069*	0.7281
*Amaryllidaceae*	29	10	11	−0.08	7	1.01E-01	0.1993	0.5281
*Anacardiaceae*	61	23	15	0.53*	14	5.87E-03*	0.2660*	0.5031
*Annonaceae*	162	66	29	1.35*	37	2.25E-07*	0.3347*	0.4845
*Apiaceae*	25	14	11	0.36	6	3.19E-04*	0.3691*	0.7341
*Apocynaceae*	226	72	37	0.97*	51	9.81E-04*	0.2613*	0.3820
*Araceae*	201	27	34	−0.19	46	1.00E+00	0.0941	0.1885
*Araliaceae*	60	23	15	0.55*	14	4.64E-03*	0.2707*	0.5104
*Arecaceae*	218	23	36	−0.35	49	1.00E+00	0.0715	0.1534
*Aristolochiaceae*	22	5	10	−0.50	5	5.81E-01	0.1023	0.4370
*Asparagaceae*	80	22	18	0.26	18	1.85E-01	0.1893	0.3819
*Aspleniaceae*	41	1	13	−0.92	9	1.00E+00	0.0058	0.1257
*Asteraceae*	237	139	39	2.67*	54	1.11E-32*	0.5228*	0.6474
*Athyriaceae*	40	1	13	−0.92	9	1.00E+00	0.0060	0.1286
*Balsaminaceae*	66	3	16	−0.81	15	1.00E+00	0.0165	0.1253
*Begoniaceae*	41	2	13	−0.84	9	1.00E+00	0.0150	0.1616
*Bignoniaceae*	42	13	13	0.04	10	1.38E-01	0.1908	0.4613
*Boraginaceae*	28	7	11	−0.35	6	4.58E-01	0.1273	0.4354
*Brassicaceae*	12	4	9	−0.54	3	2.81E-01	0.1386	0.6143
*Burseraceae*	27	4	11	−0.62	6	8.92E-01	0.0606	0.3267
*Cactaceae*	11	3	9	−0.65	2	4.72E-01	0.0992	0.5719
*Campanulaceae*	21	5	10	−0.49	5	5.36E-01	0.1073	0.4537
*Capparaceae*	35	11	12	−0.05	8	1.51E-01	0.1856	0.4811
*Celastraceae*	55	11	15	−0.23	12	7.33E-01	0.1159	0.3243
*Clusiaceae*	48	12	14	−0.10	11	4.06E-01	0.1495	0.3887
*Combretaceae*	29	21	11	0.94*	7	1.83E-08*	0.5411*	0.8527
*Commelinaceae*	40	12	13	−0.02	9	1.79E-01	0.1808	0.4554
*Connaraceae*	15	10	10	0.11	3	3.46E-04*	0.4133*	0.8480
*Convolvulaceae*	114	25	22	0.14	26	6.14E-01	0.1533	0.3040
*Crassulaceae*	10	4	9	−0.52	2	1.73E-01	0.1675	0.6921
*Cucurbitaceae*	44	27	13	1.11*	10	4.28E-08*	0.4654*	0.7430
*Cycadaceae*	14	4	9	−0.55	3	3.97E-01	0.1182	0.5510
*Cyperaceae*	254	15	40	−0.63	58	1.00E+00	0.0363	0.0952
*Davalliaceae*	18	4	10	−0.57	4	6.10E-01	0.0915	0.4557
*Dennstaedtiaceae*	18	3	10	−0.68	4	8.11E-01	0.0605	0.3958
*Dilleniaceae*	14	10	9	0.13	3	1.45E-04*	0.4490*	0.8818
*Dioscoreaceae*	38	9	12	−0.25	9	5.05E-01	0.1304	0.3933
*Dipterocarpaceae*	67	11	16	−0.30	15	9.20E-01	0.0947	0.2710
*Dryopteridaceae*	62	1	16	−0.93	14	1.00E+00	0.0039	0.0853
*Ebenaceae*	61	18	15	0.20	14	1.33E-01	0.1956	0.4195
*Elaeocarpaceae*	21	5	10	−0.49	5	5.36E-01	0.1073	0.4537
*Ericaceae*	37	1	12	−0.92	8	1.00E+00	0.0064	0.1381
*Euphorbiaceae*	226	69	37	0.89*	51	4.00E-03*	0.2489*	0.3683
*Fagaceae*	121	6	23	−0.74	27	1.00E+00	0.0234	0.1040
*Gentianaceae*	38	5	12	−0.58	9	9.53E-01	0.0586	0.2743
*Gesneriaceae*	153	5	27	−0.81	35	1.00E+00	0.0144	0.0741
*Gleicheniaceae*	10	1	9	−0.88	2	9.24E-01	0.0228	0.4128
*Hypericaceae*	11	5	9	−0.41	2	8.11E-02	0.2109	0.7233
*Icacinaceae*	12	1	9	−0.88	3	9.54E-01	0.0192	0.3603
*Lamiaceae*	233	83	38	1.22*	53	5.30E-06*	0.2975*	0.4197
*Lauraceae*	112	23	22	0.06	25	7.41E-01	0.1411	0.2896
*Lecythidaceae*	18	4	10	−0.57	4	6.10E-01	0.0915	0.4557
*Leguminosae*	530	195	77	1.57*	120	1.73E-03*	0.3280*	0.4098
*Linderniaceae*	38	10	12	−0.17	9	3.56E-01	0.1500	0.4213
*Lindsaeaceae*	23	1	11	−0.90	5	9.97E-01	0.0103	0.2112
*Loganiaceae*	19	6	10	−0.37	4	2.49E-01	0.1539	0.5428
*Loranthaceae*	31	5	12	−0.55	7	8.64E-01	0.0721	0.3279
*Lycopodiaceae*	10	3	9	−0.64	2	4.05E-01	0.1093	0.6097
*Lythraceae*	32	11	12	−0.02	7	9.00E-02	0.2040	0.5183
*Magnoliaceae*	32	3	12	−0.73	7	9.86E-01	0.0340	0.2433
*Malpighiaceae*	23	3	11	−0.70	5	9.21E-01	0.0474	0.3236
*Malvaceae*	172	63	30	1.14*	39	2.54E-05*	0.2979*	0.4406
*Marantaceae*	22	6	10	−0.39	5	3.82E-01	0.1321	0.4841
*Melastomataceae*	99	14	20	−0.30	22	9.88E-01	0.0865	0.2237
*Meliaceae*	72	20	17	0.22	16	1.86E-01	0.1877	0.3909
*Menispermaceae*	44	25	13	0.96*	10	1.06E-06*	0.4215*	0.7036
*Moraceae*	144	39	26	0.51*	33	1.25E-01	0.2050	0.3489
*Musaceae*	13	6	9	−0.31	3	5.31E-02	0.2304	0.7114
*Myristicaceae*	36	8	12	−0.32	8	5.92E-01	0.1177	0.3821
*Myrtaceae*	121	18	23	−0.21	27	9.88E-01	0.0964	0.2231
*Nepenthaceae*	12	2	9	−0.77	3	7.94E-01	0.0504	0.4545
*Nephrolepidaceae*	10	2	9	−0.76	2	7.00E-01	0.0602	0.5178
*Oleaceae*	76	13	17	−0.23	17	9.07E-01	0.1031	0.2714
*Orchidaceae*	1099	32	151	−0.79	249	1.00E+00	0.0207	0.0408
*Orobanchaceae*	20	5	10	−0.48	5	4.88E-01	0.1128	0.4717
*Oxalidaceae*	11	7	9	−0.18	2	4.25E-03*	0.3489*	0.8483
*Pandanaceae*	26	6	11	−0.43	6	5.58E-01	0.1111	0.4226
*Passifloraceae*	16	5	10	−0.45	4	2.89E-01	0.1421	0.5596
*Phyllanthaceae*	178	60	30	0.99*	40	5.25E-04*	0.2717*	0.4094
*Piperaceae*	48	12	14	−0.10	11	4.06E-01	0.1495	0.3887
*Plantaginaceae*	47	6	14	−0.54	11	9.71E-01	0.0607	0.2525
*Poaceae*	524	52	76	−0.31	119	1.00E+00	0.0765	0.1279
*Polygalaceae*	25	7	11	−0.32	6	3.34E-01	0.1433	0.4779
*Polygonaceae*	32	18	12	0.60*	7	4.16E-05*	0.3921*	0.7189
*Polypodiaceae*	128	13	24	−0.45	29	1.00E+00	0.0606	0.1662
*Primulaceae*	115	22	23	0.00	26	8.48E-01	0.1300	0.2729
*Proteaceae*	13	4	9	−0.54	3	3.39E-01	0.1276	0.5810
*Pteridaceae*	99	8	20	−0.60	22	1.00E+00	0.0420	0.1516
*Putranjivaceae*	16	1	10	−0.89	4	9.84E-01	0.0146	0.2869
*Ranunculaceae*	20	13	10	0.35	5	6.33E-05*	0.4303*	0.8189
*Rhamnaceae*	14	11	9	0.24	3	1.49E-05*	0.5191*	0.9221
*Rhizophoraceae*	17	2	10	−0.78	4	9.25E-01	0.0358	0.3471
*Rosaceae*	57	14	15	−0.03	13	4.19E-01	0.1526	0.3717
*Rubiaceae*	416	112	62	0.83*	94	2.45E-02	0.2289	0.3139
*Rutaceae*	65	34	16	1.19*	15	1.98E-07*	0.4034*	0.6401
*Salicaceae*	35	9	12	−0.22	8	3.98E-01	0.1420	0.4220
*Santalaceae*	15	2	10	−0.78	3	8.86E-01	0.0405	0.3835
*Sapindaceae*	45	18	13	0.39	10	6.97E-03*	0.2699*	0.5463
*Sapotaceae*	46	7	14	−0.46	10	9.23E-01	0.0765	0.2831
*Selaginellaceae*	20	3	10	−0.69	5	8.65E-01	0.0545	0.3634
*Smilacaceae*	29	10	11	−0.08	7	1.01E-01	0.1993	0.5281
*Solanaceae*	47	22	14	0.67*	11	2.34E-04*	0.3329*	0.6083
*Stemonaceae*	14	5	9	−0.44	3	1.94E-01	0.1634	0.6162
*Styracaceae*	10	2	9	−0.76	2	7.00E-01	0.0602	0.5178
*Symplocaceae*	17	1	10	−0.89	4	9.87E-01	0.0138	0.2729
*Tectariaceae*	14	3	9	−0.66	3	6.48E-01	0.0779	0.4809
*Theaceae*	11	2	9	−0.76	2	7.51E-01	0.0549	0.4841
*Thymelaeaceae*	12	7	9	−0.19	3	8.21E-03*	0.3158*	0.8078
*Urticaceae*	76	18	17	0.06	17	4.63E-01	0.1556	0.3441
*Verbenaceae*	13	5	9	−0.43	3	1.52E-01	0.1766	0.6486
*Violaceae*	10	3	9	−0.64	2	4.05E-01	0.1093	0.6097
*Vitaceae*	57	22	15	0.52*	13	5.05E-03*	0.2704*	0.5163
*Zingiberaceae*	290	77	45	0.72*	66	6.92E-02	0.2180	0.3192

T, total number of all species; M, number of medicinal species; P1, number of medicinal species predicted by the regression model; R, R value; P2, number of medicinal species predicted by the binomial model; Prob, binomial probability; inf., inferior of 95% Bayesian interval; sup., superior of 95% Bayesian interval. A “*” indicates that the family was estimated as MIMFs using the given method.

To investigate the geographic variation in MIMFs we used estimates of regional family diversity. In cases where the species diversity within a family was unknown for a specific region, we used instead the corresponding figure for the entire country as a conservative approach.

### Statistical Approaches

#### Linear Regression and R Values

We used regression residuals to analyze a simple plot of the number of medicinally useful species against the total number of species in a family. It should be noted that this analytical approach when used for analyzing medicinal floras typically violates the statistical assumptions of homescedasticity and normality (Bennett and Husby, 2008).

In this study, we coined a new metric that we refer to as *relative regression residual* (R). It is defined as the relative difference between the *observed number of medicinally useful species* (O) and *predicted number of medicinally useful species under the model* (P):

R=(O−P)/P

R value equals zero (0) when there is a perfect fit between predicted and observed data; values below zero (-R) implies that a given family had fewer medicinal species than expected; and values above zero (+R) implies that a family had more medicinally useful species than expected. To filter random noise, which could be problematic in data sets assembled from many sources, we introduced a critical range of R values from -0.5 to 0.5 within which we considered deviation from the model of no consequence. Families with R values above 0.5 we refer to here as MIMFs.

#### Binomial Analysis

We used the BINOMDIST function in Microsoft Excel to conduct a binomial analysis in accordance with the method described by Bennett and Husby (2008). The dataset was identical to the one used for linear regression. Families with higher numbers of medicinal species than expected from the model were identified as MIMFs. Based on the probability that the actual number of medicinal species is equal to or lower than the number of expected medicinal species (a) and the probability that the number of medicinal species is equal to the number of expected medicinal species (b), we calculated a 95% interval probability (p) that the number of medicinal species is more than the number of expected medicinal species as:

p=(1−a)+b

We considered all plant families with 95% interval probabilities less than 0.05 as MIMFs.

#### Bayesian Analysis

We conducted a Bayesian analysis according to the principles laid down by Weckerle et al. (2014) using the BETA.INV function in Microsoft Excel. We considered all plant families with an inferior 95% probability credible interval higher than the one calculated for all species in Thailand (0.2362) as MIMFs in this analysis.

## Results

### Regression Analysis

At the national scale, the modeled linear relationship between the number of medicinal species (P) and all species (T) within a given family was:

P=0.3165T+7.678

**Figure 1:**
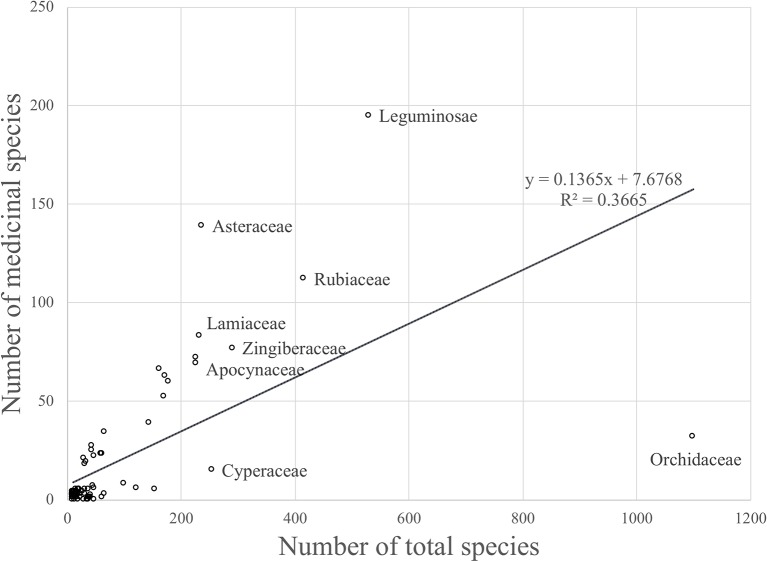
Linear regression model that shows the relationship between the number of medicinal species and the total number of species for plant families occuring in Thailand (R^2^ = 0.36646).

The model only explained 37% of the variability in the data (R^2^ = 0.37) (**Figure 1**).

Although there was a positive relationship overall between the number of medicinally useful plants within a family and the number of species it comprises, it should be noted that some of the larger families actually contained fewer medicinal plants than some of the smaller families. A number of families had no record of medicinally useful species at all and consequently scored -1 for their R values. This applied to *Thelypteridaceae*, *Hymenophyllaceae*, *Podostemaceae*, *Eriocaulaceae*, *Hydrocharitaceae*, *Burmanniaceae*, and *Cupressaceae* (**Table 1**). Using the relative regression residual (R), we identified a total of 22 MIMFs across Thailand. The families with the highest R score were in decreasing order: *Asteraceae* (R = 2.7), *Leguminosae* (R = 1.5), *Annonaceae* (R = 1.3), *Lamiaceae* (R = 1.2), *Rutaceae* (R = 1.2), and *Cucurbitaceae* (R = 1.1).

### Binomial Analysis and Bayesian Analysis

The binomial and Bayesian analyses identified the same 27 MIMFs (**Table 1**). The families *Rhamnaceae*, *Ranunculaceae*, *Dilleniaceae*, *Apiaceae*, *Connaraceae*, *Oxalidaceae*, *Sapindaceae*, and *Thymelaeaceae* were not identified by linear regression analysis as MIMFs. On the contrary, the families *Rubiaceae*, *Zingiberaceae*, and *Moraceae*, which were included in the MIMFs resulting from the linear regression analysis, were not among the MIMFs identified by the binomial and Bayesian analyses (**Table 1**).

### Combined Analyses

At the national level, 19 plant families were identified as MIMFs by all three statistical approaches (**Table 1**). Fifteen of these were shared across all villages studied Thailand: *Asteraceae*, *Leguminosae*, *Combretaceae*, *Cucurbitaceae*, *Rutaceae*, *Menispermaceae*, *Lamiaceae*, *Amaranthaceae*, *Malvaceae*, *Solanaceae*, *Phyllanthaceae*, *Apocynaceae*, *Euphorbiaceae*, *Vitaceae*, and *Acanthaceae*. The families *Annonaceae*, *Polygonaceae*, *Araliaceae*, and *Anacardiaceae* were included among the MIMFs at the national level but not shared across all villages.

### Variation in MIMFs Across Regions

The regression-residual analysis revealed substantial variation in the MIMFs across the seven regions as defined by Phumthum and Balslev (2018) (**Table 2**). The highest number of MIMFs was found in the northeastern region (27) whereas the lowest number was recorded in the southeastern region (20). In the southwestern, the northern, the central, the peninsula, and eastern regions we recorded were 25, 24, 23, 23, and 21 MIMFs, respectively. The families *Leguminosae*, *Euphorbiaceae*, *Apocynaceae*, *Malvaceae*, and *Amaranthaceae* were shared among the MIMFs of all the regions. The families *Rutaceae*, *Lamiaceae*, *Menispermaceae*, *Asteraceae*, *Combretaceae*, *Acanthaceae*, *Phyllathaceae*, and *Rubiaceae* appeared among the MIMFs in six regions, whereas *Zingiberaceae*, *Moraceae*, and *Sapindaceae* appeared in five regions. Nineteen families were recorded as MIMFs in at least four regions. The total list of MIMFs across the seven regions combined included 51 plant families.

**Table 2:** Relative regression residuals (R values) for Thai plant families in each of seven regions in Thailand as defined by Phumthum & Balslev (2018).

Family	C	E	N	NE	P	SE	SW
*Acanthaceae*	0.58*	1.13*	1.44	0.81*	0.40	2.64*	1.30*
*Achariaceae*	−0.41	−1.00	−0.69	−1.00	−1.00	−1.00	−0.27
*Amaranthaceae*	2.02*	0.79*	0.89*	1.45*	1.08*	1.41*	0.74*
*Amaryllidaceae*	−0.47	−0.53	−0.09	1.03*	−0.28	0.23	1.41*
*Anacardiaceae*	0.43	0.02	0.52*	1.37*	0.12	−1.00	1.76*
*Annonaceae*	−0.40	2.66*	−0.10	1.33	2.42*	2.72*	0.32
*Apiaceae*	0.04	−0.51	0.08	0.05	1.48*	−1.00	−0.37
*Apocynaceae*	2.40*	2.82*	0.80*	1.22*	1.37*	2.75*	1.07*
*Araceae*	−0.02	−0.38	0.03	0.01	0.43	−0.33	0.35
*Araliaceae*	−0.57	−0.31	0.7*	−0.60	0.32	1.13*	0.39
*Arecaceae*	−1.07	−0.56	−0.51	−0.24	0.53*	0.91*	−0.79
*Aristolochiaceae*	−0.45	−0.49	−0.72	−1.00	−0.49	0.27	−1.00
*Asparagaceae*	−0.13	0.17	0.6*	−0.30	−0.01	0.97*	0.61
*Aspleniaceae*	−0.51	−1.00	−0.88	−1.00	−0.78	−1.00	−1.00
*Asteraceae*	1.86*	−0.18	2.07*	0.7*	0.78*	0.82*	2.00*
*Athyriaceae*	−0.50	−1.00	−0.88	−1.00	−0.78	−1.00	−0.46
*Balsaminaceae*	−0.59	−1.00	−0.80	−0.62	−0.64	−1.00	−1.00
*Begoniaceae*	−0.51	−0.59	−0.88	−1.00	−1.00	−1.00	−1.00
*Bignoniaceae*	−0.01	0.22	0.40	−0.09	−0.36	0.16	1.68*
*Boraginaceae*	−0.47	0.42	−0.34	−0.49	−0.52	−1.00	−0.39
*Brassicaceae*	0.08	−1.00	−0.85	−0.40	−0.72	−1.00	−0.28
*Burseraceae*	0.04	−1.00	−0.47	−0.48	−1.00	−1.00	−1.00
*Cactaceae*	−0.41	−1.00	−0.69	−1.00	−0.72	−1.00	−1.00
*Campanulaceae*	−0.44	−1.00	−0.44	−0.45	−1.00	−1.00	−1.00
*Capparaceae*	0.51*	0.31	−0.01	0.44	−0.32	2.59*	−0.43
*Celastraceae*	−0.55	0.79*	−0.36	1.06*	−0.03	0.09	−0.52
*Clusiaceae*	−0.03	−1.00	−0.66	−0.57	0.64*	0.13	−1.00
*Combretaceae*	1.53*	1.34*	0.83*	2.04*	0.20	1.46*	1.41*
*Commelinaceae*	0.50*	−0.59	0.19	−0.54	−0.56	−1.00	0.63*
*Connaraceae*	−0.42	0.68*	−0.40	0.16	0.91*	−1.00	−1.00
*Convolvulaceae*	1.26*	−0.06	−0.18	−0.42	−0.04	−0.13	−0.01
*Crassulaceae*	−0.41	−1.00	−0.37	−1.00	−0.71	−1.00	−1.00
*Cucurbitaceae*	0.98*	0.19	1.76*	−0.10	0.06	0.15	2.16*
*Cycadaceae*	−0.42	−1.00	−0.40	−1.00	−1.00	−1.00	−1.00
*Cyperaceae*	−1.19	−0.74	−0.65	−0.49	−0.44	−1.00	−1.00
*Davalliaceae*	−0.43	−1.00	−0.71	−0.44	−0.47	−1.00	−1.00
*Dennstaedtiaceae*	−0.43	−1.00	−0.57	−0.44	−1.00	−1.00	−1.00
*Dilleniaceae*	−0.42	0.70*	−0.40	0.75*	0.65*	0.33	−1.00
*Dioscoreaceae*	0.00	−0.58	−0.15	−0.06	−0.11	0.18	0.11
*Dipterocarpaceae*	−0.59	−0.03	−0.51	1.28*	−0.64	0.04	−0.12
*Dryopteridaceae*	−0.58	−1.00	−0.90	−1.00	−1.00	−1.00	−1.00
*Ebenaceae*	0.93*	1.72*	0.22	0.58*	−0.25	0.06	0.8*
*Elaeocarpaceae*	−0.44	0.03	−0.86	−1.00	−0.49	−1.00	−1.00
*Ericaceae*	−0.50	−1.00	−0.88	−1.00	−1.00	−1.00	−1.00
*Euphorbiaceae*	2.40*	1.55*	1.23*	1.78*	1.54*	3.37*	1.0*
*Fagaceae*	−0.76	−1.00	−0.71	−0.72	−0.74	−1.00	−1.00
*Gelsemiaceae*	−2.07	−1.00	−0.98	−1.00	−1.00	−1.00	−1.00
*Gentianaceae*	−0.50	−0.58	−0.64	−1.00	−0.56	−1.00	−1.00
*Gesneriaceae*	−0.87	−0.62	−0.81	−1.00	−0.77	−1.00	−1.00
*Gleicheniaceae*	−0.41	−1.00	−0.84	−1.00	−1.00	−1.00	−1.00
*Hypericaceae*	−0.41	0.77*	−0.53	−0.40	−0.72	−1.00	−0.27
*Icacinaceae*	−0.42	−1.00	−0.85	−1.00	−1.00	−1.00	−1.00
*Lamiaceae*	1.38*	0.24	1.74*	1.54*	1.74*	2.68*	2.24*
*Lauraceae*	−0.74	−0.29	0.05	0.76*	0.66*	−0.13	−1.00
*Lecythidaceae*	0.56*	0.07	−0.86	−0.44	−0.21	−1.00	−1.00
*Leguminosae*	6.93*	2.27*	2.47*	2.55*	1.30*	1.49*	2.60*
*Linderniaceae*	0.00	−1.00	−0.15	−0.53	−0.33	−1.00	−0.45
*Lindsaeaceae*	−0.45	−1.00	−0.86	−1.00	−1.00	−1.00	−1.00
*Loganiaceae*	0.06	0.59*	−0.43	0.11	−1.00	0.29	−1.00
*Loranthaceae*	0.02	−0.54	−0.49	−1.00	−0.29	0.22	−1.00
*Lycopodiaceae*	−0.41	−1.00	−0.69	−1.00	−0.43	−1.00	−1.00
*Lythraceae*	−0.48	−0.10	0.02	−0.01	−0.07	−1.00	0.76*
*Magnoliaceae*	−0.48	−1.00	−0.62	−1.00	−1.00	−1.00	−1.00
*Malpighiaceae*	−0.45	−1.00	−0.59	−1.00	−1.00	−1.00	−1.00
*Malvaceae*	6.07*	1.62*	1.24*	1.02*	0.80*	3.33*	1.02*
*Marantaceae*	0.05	−1.00	−0.44	−0.46	−0.23	−1.00	−1.00
*Melastomataceae*	−0.69	0.03	−0.44	−0.37	0.04	−0.08	−1.00
*Meliaceae*	−0.61	−0.07	0.23	−0.27	0.39	−1.00	0.28
*Menispermaceae*	0.48	1.39*	1.30*	1.69*	1.11*	4.73*	2.16*
*Moraceae*	1.16*	0.20	0.68*	0.01	0.55*	2.14*	1.28*
*Musaceae*	−0.42	−1.00	−0.24	0.77	−0.16	−1.00	−0.29
*Myristicaceae*	−0.49	−0.14	−0.63	−1.00	0.13	0.19	−1.00
*Myrtaceae*	−0.76	−0.10	−0.35	−0.44	0.19	−1.00	−0.36
*Nepenthaceae*	−0.42	−1.00	−1.00	−1.00	−0.44	−1.00	−1.00
*Nephrolepidaceae*	−0.41	−1.00	−0.69	−1.00	−1.00	−1.00	−1.00
*Oleaceae*	0.38	0.51*	−0.36	−1.00	−0.15	0.00	−1.00
*Orchidaceae*	−3.89	−0.72	−0.86	−0.95	−0.77	−1.00	−0.79
*Orobanchaceae*	−0.44	−0.48	−0.72	−0.45	−0.74	−1.00	−1.00
*Oxalidaceae*	1.09*	−1.00	0.09	−1.00	−0.43	−1.00	−1.00
*Pandanaceae*	−0.46	−0.52	−0.73	−0.48	0.47	0.25	−0.38
*Passifloraceae*	0.07	−0.45	−0.70	−1.00	−0.46	−1.00	0.38
*Phyllanthaceae*	2.55*	1.73*	1.25*	1.19*	1.90*	0.42	0.97
*Piperaceae*	−0.03	−1.00	0.23	0.30	0.44	2.37*	1.54*
*Plantaginaceae*	−0.03	−0.23	−0.66	−0.56	−0.17	0.13	−0.49
*Poaceae*	−2.05	−0.66	−0.03	0.23	−0.15	−1.00	−0.48
*Polygalaceae*	0.04	0.47	−0.46	−1.00	−0.50	−1.00	−1.00
*Polygonaceae*	0.52*	−1.00	0.9*	0.48	−0.07	−1.00	−1.00
*Polypodiaceae*	−0.79	−0.78	−0.37	−0.46	−0.10	−1.00	−0.69
*Primulaceae*	−0.24	−0.30	0.26	−0.14	−0.05	−0.14	−0.67
*Proteaceae*	−0.42	−0.43	−0.70	−1.00	−1.00	−1.00	−0.29
*Pteridaceae*	−0.69	−1.00	−0.60	−1.00	−0.41	−1.00	−1.00
*Putranjivaceae*	−0.43	−1.00	−1.00	−0.43	−1.00	−1.00	−0.31
*Ranunculaceae*	−0.44	−1.00	0.70*	−1.00	−0.74	−1.00	−1.00
*Rhamnaceae*	0.58*	−0.43	0.66*	1.92*	−0.72	0.33	−0.29
*Rhizophoraceae*	−0.43	−0.46	−1.00	−0.43	−0.46	0.31	−1.00
*Rosaceae*	−0.56	−0.65	0.25	−0.19	−0.62	−1.00	−1.00
*Rubiaceae*	1.79*	1.62*	0.74*	0.8*	1.34*	1.54*	0.14
*Rutaceae*	−0.58	3.61*	1.27*	2.08*	1.18*	6.32*	3.91*
*Salicaceae*	−0.49	−0.13	−0.26	0.44	−0.77	−1.00	−0.43
*Santalaceae*	−0.42	−0.44	−0.70	−0.42	−1.00	−1.00	−1.00
*Sapindaceae*	2.48*	0.18	0.03	1.66*	0.68*	2.42*	1.09*
*Sapotaceae*	−0.52	−1.00	−0.55	−0.56	−0.38	−1.00	−0.48
*Selaginellaceae*	−0.44	−1.00	−0.57	−1.00	−1.00	−1.00	−1.00
*Smilacaceae*	−0.47	−0.07	0.04	−1.00	−0.04	0.23	−1.00
*Solanaceae*	0.47	−0.23	1.03*	2.06*	0.86*	0.13	4.13*
*Stemonaceae*	−0.42	−0.43	−0.40	−0.42	−0.45	−1.00	−0.29
*Styracaceae*	−0.41	−0.40	−0.69	−1.00	−1.00	−1.00	−1.00
*Symplocaceae*	−0.43	−0.46	−1.00	−1.00	−1.00	−1.00	−1.00
*Tectariaceae*	−0.42	−1.00	−0.70	−1.00	−1.00	0.33	−0.29
*Theaceae*	−0.41	−1.00	−0.69	−1.00	−1.00	−1.00	−1.00
*Thymelaeaceae*	−0.42	−0.42	−1.00	0.79*	0.41	0.34	−0.28
*Urticaceae*	0.38	−1.00	0.47	−1.00	−0.66	0.00	−0.59
*Verbenaceae*	0.58*	−1.00	−0.54	−0.41	0.11	0.34	0.43
*Violaceae*	−0.41	−1.00	−0.69	−1.00	−1.00	−1.00	−0.26
*Vitaceae*	−0.06	2.87*	0.67*	0.22	0.54*	−1.00	0.42
*Zingiberaceae*	−0.80	0.73*	0.61*	0.99*	1.03*	−0.46	1.73*

C, central; E, eastern; N, northern; NE, northeastern; P, peninsula; SE, southeastern and SW,southwestern. A “*” indicates that the family was estimated as MIMF using this method.

The binomial and Bayesian analyses identified the 44 MIMFs in total at the regional level. None of these families were shared across all seven regions. *Rutaceae*, *Leguminosae*, *Menispermaceae*, *Euphorbiaceae*, and *Combretaceae* were identified as MIMFs in six regions; *Sapindaceae*, *Amaranthaceae*, and *Solanaceae*, in five regions; *Lamiaceae*, *Malvaceae*, and *Phyllanthaceae*, in four regions (**Table 3**).

**Table 3:** Binomial probabilities of the plant families with higher numbers of medicinal species than expected under the null model of a perfect relationship with family size.

Family	C	E	N	NE	P	SE	SW
*Acanthaceae*	0.488	0.055	0.000*	0.24	0.490	0.061	0.070
*Achariaceae*	1.000	1.000	0.455	1.00	1.000	1.000	0.285
*Amaranthaceae*	0.000*	0.049*	0.000*	0.001*	0.001*	0.063	0.076
*Amaryllidaceae*	1.000	0.713	0.091	0.002*	0.367	0.304	0.011*
*Anacardiaceae*	0.244	0.477	0.016	0.002*	0.299	1.000	0.010*
*Annonaceae*	0.920	0.000*	0.965	0.06	0.000*	0.052	0.537
*Apiaceae*	0.323	0.659	0.015	0.22	0.000*	1.000	0.533
*Apocynaceae*	0.064	0.000*	0.102	0.10	0.010*	0.064	0.145
*Araceae*	0.820	0.972	0.954	0.84	0.531	0.919	0.562
*Araliaceae*	1.000	0.725	0.002*	0.880	0.156	0.171	0.269
*Arecaceae*	1.000	0.995	1.000	0.95	0.454	0.508	0.999
*Aristolochiaceae*	1.000	0.612	0.823	1.00	0.494	0.240	1.000
*Asparagaceae*	0.713	0.436	0.021	0.78	0.551	0.261	0.219
*Aspleniaceae*	1.000	1.000	0.998	1.00	0.958	1.000	1.000
*Asteraceae*	0.165	0.936	0.000*	0.32	0.232	0.564	0.006*
*Athyriaceae*	1.000	1.000	0.997	1.00	0.954	1.000	0.704
*Balsaminaceae*	1.000	1.000	0.999	0.91	0.961	1.000	1.000
*Begoniaceae*	1.000	0.829	0.998	1.00	1.000	1.000	1.000
*Bignoniaceae*	0.481	0.259	0.009	0.44	0.613	0.408	0.008*
*Boraginaceae*	1.000	0.112	0.334	0.63	0.626	1.000	0.574
*Brassicaceae*	0.171	1.000	0.828	0.35	0.604	1.000	0.306
*Burseraceae*	0.344	1.000	0.514	0.62	1.000	1.000	1.000
*Cactaceae*	1.000	1.000	0.455	1.00	0.572	1.000	1.000
*Campanulaceae*	1.000	1.000	0.321	0.53	1.000	1.000	1.000
*Capparaceae*	0.102	0.182	0.096	0.12	0.488	0.009*	0.656
*Celastraceae*	1.000	0.081	0.780	0.004*	0.389	0.497	0.813
*Clusiaceae*	0.528	1.000	0.968	0.82	0.024*	0.451	1.000
*Combretaceae*	0.001*	0.007*	0.000*	0.000*	0.060	0.050*	0.011*
*Commelinaceae*	0.128	0.821	0.039	0.76	0.808	1.000	0.118
*Connaraceae*	1.000	0.023*	0.138	0.10	0.000*	1.000	1.000
*Convolvulaceae*	0.102	0.712	0.922	0.91	0.751	0.759	0.668
*Crassulaceae*	1.000	1.000	0.037	1.00	0.538	1.000	1.000
*Cucurbitaceae*	0.031*	0.283	0.000*	0.46	0.227	0.422	0.002*
*Cycadaceae*	1.000	1.000	0.113	1.00	1.000	1.000	1.000
*Cyperaceae*	1.000	1.000	1.000	0.99	1.000	1.000	1.000
*Davalliaceae*	1.000	1.000	0.726	0.47	0.391	1.000	1.000
*Dennstaedtiaceae*	1.000	1.000	0.454	0.47	1.000	1.000	1.000
*Dilleniaceae*	1.000	0.019*	0.113	0.001*	0.000*	0.160	1.000
*Dioscoreaceae*	0.448	0.805	0.257	0.39	0.312	0.378	0.316
*Dipterocarpaceae*	1.000	0.540	0.961	0.003*	0.964	0.567	0.601
*Dryopteridaceae*	1.000	1.000	1.000	1.00	1.000	1.000	1.000
*Ebenaceae*	0.069	0.004*	0.121	0.17	0.673	0.533	0.111
*Elaeocarpaceae*	1.000	0.221	0.954	1.00	0.469	1.000	1.000
*Ericaceae*	1.000	1.000	0.996	1.00	1.000	1.000	1.000
*Euphorbiaceae*	0.064	0.008*	0.002*	0.001*	0.003*	0.024*	0.009*
*Fagaceae*	1.000	1.000	1.000	0.99	0.999	1.000	1.000
*Gelsemiaceae*	1.000	1.000	1.000	1.00	1.000	1.000	1.000
*Gentianaceae*	1.000	0.805	0.907	1.00	0.785	1.000	1.000
*Gesneriaceae*	1.000	0.989	1.000	1.00	1.000	1.000	1.000
*Gleicheniaceae*	1.000	1.000	0.770	1.00	1.000	1.000	1.000
*Hypericaceae*	1.000	0.010*	0.182	0.32	0.572	1.000	0.285
*Icacinaceae*	1.000	1.000	0.828	1.00	1.000	1.000	1.000
*Lamiaceae*	0.295	0.650	0.000*	0.004*	0.001*	0.072	0.002*
*Lauraceae*	1.000	0.855	0.680	0.20	0.128	0.753	1.000
*Lecythidaceae*	0.031*	0.174	0.929	0.47	0.145	1.000	1.000
*Leguminosae*	0.002*	0.000*	0.000*	0.000*	0.026	0.485	0.000*
*Linderniaceae*	0.448	1.000	0.257	0.74	0.544	1.000	0.686
*Lindsaeaceae*	1.000	1.000	0.966	1.00	1.000	1.000	1.000
*Loganiaceae*	0.257	0.044*	0.256	0.14	1.000	0.211	1.000
*Loranthaceae*	0.384	0.736	0.628	1.00	0.408	0.321	1.000
*Lycopodiaceae*	1.000	1.000	0.406	1.00	0.167	1.000	1.000
*Lythraceae*	1.000	0.392	0.062	0.31	0.211	1.000	0.070
*Magnoliaceae*	1.000	1.000	0.832	1.00	1.000	1.000	1.000
*Malpighiaceae*	1.000	1.000	0.626	1.00	1.000	1.000	1.000
*Malvaceae*	0.000*	0.006*	0.001*	0.15	0.141	0.021*	0.147
*Marantaceae*	0.291	1.000	0.354	0.54	0.222	1.000	1.000
*Melastomataceae*	1.000	0.603	0.987	0.87	0.609	0.709	1.000
*Meliaceae*	1.000	0.589	0.178	0.72	0.165	1.000	0.367
*Menispermaceae*	0.149	0.010*	0.000*	0.000*	0.001*	0.000*	0.002*
*Moraceae*	0.186	0.568	0.082	0.75	0.273	0.105	0.069
*Musaceae*	1.000	1.000	0.024	0.001*	0.067	1.000	0.327
*Myristicaceae*	1.000	0.451	0.886	1.00	0.126	0.362	1.000
*Myrtaceae*	1.000	0.754	0.989	0.93	0.548	1.000	0.881
*Nepenthaceae*	1.000	1.000	1.000	1.00	0.223	1.000	1.000
*Nephrolepidaceae*	1.000	1.000	0.406	1.00	1.000	1.000	1.000
*Oleaceae*	0.330	0.216	0.909	1.00	0.674	0.613	1.000
*Orchidaceae*	1.000	1.000	1.000	1.00	1.000	1.000	1.000
*Orobanchaceae*	1.000	0.577	0.779	0.51	0.786	1.000	1.000
*Oxalidaceae*	0.001*	1.000	0.000*	1.00	0.195	1.000	1.000
*Pandanaceae*	1.000	0.673	0.888	0.61	0.011*	0.277	0.547
*Passifloraceae*	0.221	0.498	0.663	1.00	0.336	1.000	0.082
*Phyllanthaceae*	0.022*	0.004*	0.001*	0.10	0.000*	0.649	0.168
*Piperaceae*	0.528	1.000	0.056	0.24	0.063	0.021*	0.014*
*Plantaginaceae*	0.520	0.594	0.964	0.81	0.466	0.444	0.761
*Poaceae*	1.000	1.000	1.000	0.93	1.000	1.000	1.000
*Polygalaceae*	0.323	0.086	0.451	1.00	0.564	1.000	1.000
*Polygonaceae*	0.088	1.000	0.000*	0.10	0.211	1.000	1.000
*Polypodiaceae*	1.000	0.996	0.994	0.94	0.846	1.000	0.980
*Primulaceae*	0.834	0.867	0.403	0.77	0.759	0.762	0.970
*Proteaceae*	1.000	0.428	0.547	1.00	1.000	1.000	0.327
*Pteridaceae*	1.000	1.000	0.998	1.00	0.942	1.000	1.000
*Putranjivaceae*	1.000	1.000	1.000	0.44	1.000	1.000	0.386
*Ranunculaceae*	1.000	1.000	0.000*	1.00	0.786	1.000	1.000
*Rhamnaceae*	0.019*	0.452	0.000*	0.000*	0.661	0.160	0.347
*Rhizophoraceae*	1.000	0.519	1.000	0.46	0.364	0.191	1.000
*Rosaceae*	1.000	0.914	0.081	0.60	0.932	1.000	1.000
*Rubiaceae*	0.466	0.002*	0.399	0.39	0.019*	0.412	0.878
*Rutaceae*	1.000	0.000*	0.000*	0.000*	0.003*	0.000*	0.000*
*Salicaceae*	1.000	0.437	0.342	0.12	0.933	1.000	0.656
*Santalaceae*	1.000	0.475	0.627	0.41	1.000	1.000	1.000
*Sapindaceae*	0.000*	0.294	0.153	0.000*	0.017*	0.018*	0.046*
*Sapotaceae*	1.000	1.000	0.890	0.81	0.674	1.000	0.754
*Selaginellaceae*	1.000	1.000	0.527	1.00	1.000	1.000	1.000
*Smilacaceae*	1.000	0.347	0.036	1.00	0.165	0.304	1.000
*Solanaceae*	0.165	0.594	0.000*	0.000*	0.007*	0.444	0.000*
*Stemonaceae*	1.000	0.452	0.113	0.39	0.279	1.000	0.347
*Styracaceae*	1.000	0.350	0.406	1.00	1.000	1.000	1.000
*Symplocaceae*	1.000	0.519	1.000	1.00	1.000	1.000	1.000
*Tectariaceae*	1.000	1.000	0.589	1.00	1.000	0.160	0.347
*Theaceae*	1.000	1.000	0.455	1.00	1.000	1.000	1.000
*Thymelaeaceae*	1.000	0.403	1.000	0.001*	0.001*	0.139	0.306
*Urticaceae*	0.330	1.000	0.050	1.00	0.980	0.613	0.901
*Verbenaceae*	0.017*	1.000	0.258	0.37	0.013*	0.150	0.056
*Violaceae*	1.000	1.000	0.406	1.00	1.000	1.000	0.263
*Vitaceae*	0.590	0.000*	0.003*	0.32	0.059	1.000	0.244
*Zingiberaceae*	0.989	0.244	0.432	0.22	0.095	0.973	0.015*

C, central; E, eastern; N, northern; NE, northeastern; P, peninsula; SE, southeastern and SW, southwestern. A “*” indicates that the family was estimated as MIMF using this method.

## Discussion

### Combining Statistical Tools in the Evaluation of the Medicinal Importance of Plant Families

The three statistical approaches used here to evaluate MIMFs at the national and regional levels in Thailand have all been used previously for analyzing medicinal floras in other parts of the world, but never in combination. Similar to Bennett and Husby (2008) and Weckerle et al. (2014), we found that the Binomial and Bayesian analyses are more sensitive to small families. By using these two statistical methods, we added the plant families *Rhamnaceae* (14 spp.), *Ranunculaceae* (20 spp.), *Dilleniaceae* (14 spp.), *Apiaceae* (25 spp.), *Connaraceae* (15 spp.), *Oxalidaceae* (11 spp.), *Sapindaceae* (45 spp.), and *Thymelaeaceae* (12 spp.) to the list of MIMFs (**Table 1**). Interestingly, the R values were biased toward the larger families and identified *Zingiberaceae* (290 spp.) and *Rubiaceae* (416 spp.) as MIMFs, whereas the Binomial and Bayesian analyses did not (**Table 1**). This result confirms previous findings that these two families are high in numbers of medicinal use reports (691 and 608 respectively) and in use value scores (5.71 and 5.02, respectively) (Phumthum et al., 2018).

Despite the fact that the three statistical approaches applied here to estimate medicinal importance of plant families all have their strength and weaknesses, our results show remarkable overlap in the results (**Table 1**). This applies especially to the national scale where we obtained robust estimates of the MIMFs due to large sample sizes. In cases where sample sizes are moderate, e.g., at smaller spatial scales, it is particularly important to combine statistical approaches to avoid inherent methodological biases.

The number of MIMFs identified by the three statistical approaches varied between 20 and 27 across the regions (**Table 2**). Of the three methodological approaches, the binomial analysis generally identified fewer MIMFs (8–20). The highest number was recovered in the northern region, the lowest in the southeastern region. The fact that none of the families were estimated as MIMF throughout all regions (**Table 3**) is an artifact of missing information, *viz*., when the species diversity of a family was unknown for a specific region, we substituted it with the family diversity for the entire country. Since we used the same datasets throughout the analyses, this cannot, however, explain the differences between the three analytical approaches. The use of R values at the regional scale interestingly resulted in uniform results across scales, as for both the number and overlap of MIMFs are concerned (**Tables 1** and **2**).

### Most Important Medicinal Families (MIMFs)

At the national scale, the combined statistical analyses identified 19 plant families as being MMIFs: *Asteraceae*, *Leguminosae*, *Combretaceae*, *Cucurbitaceae*, *Rutaceae*, *Annonaceae*, *Menispermaceae*, *Lamiaceae*, *Amaranthaceae*, *Malvaceae*, *Polygonaceae*, *Solanaceae*, *Phyllanthaceae*, *Apocynaceae*, *Euphorbiaceae*, *Araliaceae*, *Vitaceae*, *Anacardiaceae*, and *Acanthaceae* (**Table 1**). Many of these families have also been identified as MIMFs in other Asian countries such as Nepal (Saslis-Lagoudakis et al., 2011.) and South Korea (Moerman and Pemberton, 1999). The Nepali folk medicine shares most MIMFs with Thailand: *Malvaceae*, *Lamiaceae*, *Apocynaceae*, *Asteraceae*, *Cucurbitaceae*, and *Euphorbiaceae*. This is most probably due to the fact that the Nepali and the northern Thai flora contain some of the same phytogeographic elements. The flora of South Korea, which is located at subtropical and temperate latitudes, is different. Although some overlap with Thai MIMFs such as *Lamiaceae*, *Apocynaceae*, or *Leguminosae*, many temperate families such as *Aceraceae*, *Campanulaceae*, and *Ericaceae* are medicinally important in the South Korean folk medicine (**Table 4**).

**Table4:** Important medicinal families identified in ethnomedicinal studies from other parts of the world.

Family	North America I	Nepal	South Africa Cape	New Zealand	Beliz I	Mexico	Beliz II	Southern Africa	North America II	Korea	Guatamala	India	Ecuador I	Italy	Ecuador II	Hawaii
*Acanthaceae*					*											
*Aceraceae*									*	*						
*Adiantaceae*					*											
*Amaranthaceae*												*			*	
*Amaryllidaeceae*			*													
*Anacardiaceae*		*	*					*	*		*					
*Apiaceae*	*								*	*	*				*	
*Apocynaceae*		*						*		*	*		*	*		
*Araceae*					*		*			*			*			
*Araliaceae*				*					*	*	*					
*Arecaceae*													*			
*Asparagaceae*			*													
*Asteraceae*	*	*	*	*	*	*	*	*	*		*	*				
*Betulaceae*									*							
*Bignoniaceae*													*			
*Boraginaceae*								*			*		*			*
*Brassicaceae*											*					
*Campanulaceae*										*						
*Caprifoliaceae*	*								*		*			*		
*Caryophyllaceae*				*							*					
*Celastraceae*			*													
*Clethraceae*											*					
*Clusiaceae*		*														
*Combretaceae*		*														
*Commelinaceae*											*					
*Convolvulaceae*		*	*					*		*						*
*Cornaceae*									*							
*Costaceae*															*	
*Cucurbitaceae*		*												*		
*Cyclanthaceae*													*			
*Dioscoreaceae*											*					*
*Ebenaceae*			*													
*Elaeagnaceae*									*							
*Ericaceae*	*								*	*	*					*
*Euphorbiaceae*		*				*		*		*		*	*			
*Fagaceae*	*									*	*					
*Gentianaceae*										*	*					
*Geraniaceae*			*	*						*						
*Gesneriaceae*													*			
*Grossulariaceae*									*							
*Iridaceae*										*	*					
*Juglandaceae*									*							
*Lamiaceae*	*	*	*			*			*	*	*			*	*	
*Lauraceae*										*						
*Leguminosae*		*				*	*	*		*						
*Liliaceae*										*	*					
*Loranthaceae*													*	*		
*Lythraceae*		*									*					
*Magnoliaceae*									*							
*Malpighiaceae*													*			
*Malvaceae*		*	*	*				*					*	*		*
*Melanthaceae*														*		
*Melastomataceae*		*					*									
*Meliaceae*											*					
*Menispermaceae*													*			
*Monimiaceae*					*								*			
*Moraceae*		*								*				*		*
*Myricaceae*									*							
*Myrtaceae*				*												*
*Nyctaginaceae*											*					
*Oleaceae*		*														
*Onagraceae*										*	*					
*Orobanchaceae*									*							
*Pandanaceae*																*
*Papaveraceae*										*						
*Pedaliaceae*									*							
*Pinaceae*	*															
*Piperaceae*					*	*	*								*	
*Plantaginaceac*									*							
*Plumbaginaceae*									*							*
*Poaceae*			*													*
*Podocarpaceae*				*												
*Polygonaceae*		*										*				
*Primulaceae*														*		
*Ranunculaceae*	*								*	*	*	*				
*Rhamnaceae*											*					
*Rosaceae*	*	*							*		*			*		*
*Rubiaceae*		*	*		*		*	*								
*Rutaceae*		*								*						
*Salicaceae*	*								*					*		
*Sapindaceae*																*
*Smilacaceae*									*							*
*Solanaceae*		*	*		*			*			*	*		*		*
*Symplocaceae*		*														
*Urticaceae*		*											*	*		
*Valerianaceae*											*					
*Verbenaceae*		*			*					*	*		*			
*Violaceae*														*		
*Vitaceae*		*							*							
*Zingiberaceae*		*											*		*	*

**Data sources**: North America I (Moerman, 1996), Nepal, South African Cape, New Zealand (Saslis-Lagoudakis et al., 2011), Beliz I (Bourbonnas-Spear et al., 2005), Mexico (Leonti et al., 2003), Beliz II (Amiguet et al., 2005), South Africa (Douwes et al., 2008), North America II (Moerman and Pemberton 1999), Korea (Moerman and Pemberton 1999), Guatemala (Moerman and Pemberton 1999), India (Moerman and Pemberton 1999), Ecuador I (Moerman and Pemberton 1999), Italy (Weckerle et al., 2011), Ecuador II (Bennett and Husby 2008), and Hawaii (Ford and Gaoue 2017).

Within Thailand, it is noticeable that the list of MIMFs from the different regions is so similar despite the fact that the regional floras are composed of different phytogeographic elements. This is particularly obvious, when the northern and the peninsular regions are compared. The northern region contains a Indo-Burmese phytogeographic element, whereas the peninsular region has a pronounced Malayan element (van Welzen et al., 2011). Fifteen plan families were shared between the list of MIMFs identified from northern region (27) and the peninsular region (23), respectively: *Leguminosae*, *Euphorbiaceae*, *Apocynaceae*, *Malvaceae*, *Amaranthaceae*, *Rutaceae*, *Lamiaceae*, *Menispermaceae*, *Asteraceae*, *Phyllanthaceae*, *Rubiaceae*, *Zingiberaceae*, *Moraceae*, *Solanaceae*, and *Vitaceae*. This corresponds to a 60% overlap (**Table 2**). Our results indicate that although there are pronounced regional differences in the flora throughout Thailand, the MIMFs remain remarkably similar.

At the global scale, at least 90 plant families were cited as most important across 16 studies conducted in North America, Nepal, South Africa, New Zealand, Belize, Mexico, South Korea, Guatemala, India, Ecuador, Italy, and Hawaii (**Table 4**). Of these, 21 families were identified as most important repeatedly in at least four countries. Those were *Asteraceae* (11 countries), *Lamiaceae* (9), *Solanaceae* (8), *Malvaceae* (7), *Apocynaceae* (6), *Euphorbiaceae* (6), *Rosaceae* (6), *Anacardiaceae* (5), *Apiaceae* (5), *Leguminosae* (5), *Convolvulaceae* (5), *Ericaceae* (5), *Ranunculaceae* (5), *Rubiaceae* (5), *Verbenaceae* (5), *Araliaceae* (4), *Moraceae* (4), *Piperaceae* (4), *Zingiberaceae* (4), *Araceae* (4), *Boraginaceae* (4), and *Caprifoliaceae* (4) (**Table 4**). On this background, it is interesting that families of global medicinal importance such as *Asteraceae*, *Leguminosae*, *Rutaceae*, *Lamiaceae*, *Malvaceae*, *Solanaceae*, *Apocynaceae*, *Euphorbiaceae*, *Araliaceae*, and *Anacardiaceae* are also found among the MIMFs both at the national and regional scales in Thailand (**Tables 1**, **2**, and **4**). Shared knowledge concerning the effect of certain plant species on the human physiology have either been discovered independently or as a result of cultural exchange. To confirm this, we need detailed, comparative anthropological studies through space and time.

## Conclusion

This study fills a gap in our ethnomedicinal knowledge about Thailand and the Southeast Asian region in general. By using three different analytical approaches, we recovered the MIMFs in Thailand and revealed substantial differences at the regional level. Ten of the MIMFs in Thailand are also listed as important elsewhere in the world: *Asteraceae*, *Leguminosae*, *Rutaceae*, *Lamiaceae*, *Malvaceae*, *Solanaceae*, *Apocynaceae*, *Euphorbiaceae*, *Araliaceae*, and *Anacardiaceae*. We consider these families particularly promising for the pharmacological industry because their widespread use is most probably due to their content of physiologically active compounds.

We recommend that statistical approaches are combined especially when dealing with small to moderate sample sizes to avoid inherent methodological biases. It should be noted that regression residuals as used in this study to identify MIMFs apparently are less sensitive to artifacts caused by unequal family sizes. Differences in the MIMFs identified across the regions in Thailand are determined by both floristic and cultural factors. Recovering ethnomedicinal usage patterns in Thailand and elsewhere will inform natural resource management and guide pharmacologists in their constant search for chemical compound with hitherto, unknown physiological effects, which can be used as a starting point for developing new drugs.

## Data Availability Statement

All datasets generated for this study are included in the manuscript/supplementary files.

## Author Contributions

MP conceived the idea, collected and analyzed data, and wrote the manuscript. HB supervised the research and wrote the manuscript. AB supported the analyses, wrote the manuscript, and supervised the research.

## Funding

The study was supported by a grant to HB from the Carlsberg Foundation to the Flora of Thailand research project.

## Conflict of Interest

The authors declare that the research was conducted in the absence of any commercial or financial relationships that could be construed as a potential conflict of interest.
